# Dog‐assisted therapy in the dental clinic. Part B. Hazards and assessment of potential risks to the health and safety of the dental therapy dog

**DOI:** 10.1002/cre2.239

**Published:** 2019-08-20

**Authors:** Anne M. Gussgard, J. Scott Weese, Arne Hensten, Asbjørn Jokstad

**Affiliations:** ^1^ Faculty of Health Sciences UiT The Arctic University of Norway Tromsø Norway; ^2^ Centre for Public Health and Zoonoses, Ontario Veterinary College University of Guelph Guelph Ontario Canada

**Keywords:** animal occupational injuries, behaviour, dental clinics, dogs

## Abstract

**Background:**

A dental therapy dog may help anxious patients in the dental clinic overcome their fear and facilitate the completion of necessary dental care. Dental clinic activities are associated with hazards that may pose potential risks to the health and safety of the dental therapy dog.

**Objectives:**

To describe potential hazards associated with risks to health and safety to therapy dogs in dental clinics and to present suggestions for risk minimisation by adopting best practices in dental clinic settings.

**Materials and method:**

Literature searches in Medline, http://Clinicaltrials.gov, and Google Scholar for qualitative and quantitative assessments of occupational hazards and risks in dental clinics, in combination with a review of the reference list of the included studies. Identified hazards and risks were analysed relative to their relevance for the health and welfare of a therapy dog present in a dental clinic setting.

**Results:**

Workplace hazards in the dental clinic that apply to both humans and therapy dogs are allergies, sharps injury, eye injury, stress, rhinitis, hearing impairment, and other hazards. Additional concerns associated with risks for the dental therapy dog are situations involving erratic patient behaviour and threats if the patient is an undisclosed disease carrier. Risks to the health and safety of the dental therapy dog in the clinics are present but are low if the dental clinical staff and dog handlers comply with best practices.

**Conclusions:**

Best practice includes awareness amongst the clinic staff and the dog handler of all potential hazards in the dental clinic and on how to reduce these hazards as well as adverse events that may scare the dental therapy dog. The dental therapy dog team must be specially trained to work in a dental clinic. Each treatment session has to be exclusively tailored to that specific appointment and the individual patient.

## 1 INTRODUCTION

Dental anxiety and fear are common, and estimates of prevalence range between 15% and 35% in the literature, depending on the study population and tool used for measuring anxiety and fear. Clinicians have attempted a wide range of antianxiety approaches to managing patients, albeit with varying success or added risks of adverse events. Conventional behaviour management techniques that aim to establish a positive relationship between the clinician and patient include but are not limited to positive reinforcement, voice control, non‐verbal communication, and tell–show–do (Oliver & Manton, 2015). Distraction techniques may include audio, audio‐visual, modelling, instrument camouflage, biofeedback, dental‐operating microscope, or toys (Prado et al., 2019). Patient management strategies may be time‐consuming, and effectiveness studies are inconsistent regarding reported reduced anxiety or intended behavioural changes. Sedation is also used to manage the behaviour of patients undergoing dental treatment, although finding the optimal sedation agent remains elusive. For children, at least 34 different sedative agents with or without a combination of inhalation of nitrous oxide have been evaluated in randomised controlled trials, but only orally administrated midazolam appears to be both effective and safe (Ashley, Chaudhary, & Lourenço‐Matharu, 2018). Many countries allow sedation methods just to be used in paediatric clinics with authorised specialists due to safety reasons. The same applies to the use of dental treatment under general anaesthesia, which involves even higher risks. Moreover, recent studies suggest that patient anxiety does not decrease afterwards (Haworth, Dudding, Waylen, Thomas, & Timpson, 2017). An emerging promising alternative for anxious patients is the use of a dental therapy dog (Derosier, 2016; Gupta & Yadav, 2018; Manley, 2016; Nammalwar & Rangeeth, 2018). The basis for the claims that the presence of a dental therapy dog in the clinic is effective is currently largely anecdotal. There is no scientific data yet that convincingly establish that a therapy dog decreases patient anxiety in the dental chair. Nevertheless, given the higher risk procedures associated with sedation or general anaesthesia, there is an interest to pursue alternative approaches to managing anxious patients.

To the authors' knowledge, there are no publications that describe the legal aspects of trained dental therapy dogs working in dental clinics, in context with occupational safety and health regulations.

There is a need to systematically identify all potential hazards associated with implementing dog‐assisted therapy in a dental clinic that may be related to risk to health and safety, estimate the likelihood of adverse events, and guide how to minimise and control risks for the patients, the dentist, and the clinic staff. However, also the welfare of the dental therapy dog must be secured. In addition to the multitude of workplace hazards in the dental clinic that applies to both humans and animals, additional concerns associated with risks for the dental therapy dog are situations involving erratic patient behaviour, as well as risks if the patient is an undisclosed disease vector.

The objective of the current paper is to describe potential hazards associated with risks to health and safety to the dental therapy dog in dental clinics and to present suggestions for risk minimisation by adopting proposed best practices in dental clinic settings. Hazards and assessment of potential risks to the health and safety of humans are described in a parallel article (Gussgard, Weese, Hensten, & Jokstad, 2019).

## 2 MATERIALS AND METHODS

The authors performed literature searches in Medline, http://Clinicaltrials.gov, and Google Scholar for qualitative and quantitative assessments of occupational hazards and risks in dental clinic settings, in combination with a review of the reference list of the included studies. The search strategy in Medline through http://Pubmed.com was ((“occupational dentistry”[MeSH Terms] OR (“occupational”[All Fields] AND “dentistry”[All Fields]) OR “occupational dentistry”[All Fields]) and filtered for reviews. No time limitation or language filters were used, yielding *n* = 405 articles. Combining the search strategy with the search strategy used to identify dog‐assisted therapy described in the parallel paper (Gussgard et al., 2019) yielded no articles. Hence, the hazards and risks to the health and welfare for humans in a dental clinic setting were critically appraised with respect to their potential relevance to the health and safety for a dental therapy dog working in a dental clinic setting.

## 3 RESULTS

### 3.1 Workplace hazards in a dental clinic setting

Dental clinical practice has always been associated with hazards for the clinic staff members, despite many changes in exposure to materials, therapeutic techniques, equipment, ergonomic designs and protocols to avoid cross‐infection and radiological protective protocols (Hensten‐Pettersen & Jacobsen, 1990; Moodley, Naidoo, & Wyk, 2018).

Prevailing hazards associated with risks for occupational health problems for dental clinic staff members apply also to a dental therapy dog. Currently, there are no data to substantiate a ranking of their relevance regarding risks of health problems for the dental therapy dog (Table 1). We have chosen to present and discuss the occupational hazards in the sequence of importance as they apply to humans, acknowledging that this sequence may in due time be shown to be different for dental therapy dogs.

**Table 1:** Hazards in the dental clinic ranked by frequency of reported occupational health problems amongst clinic staff workers

Health problem	Hazards/aetiology
1. Allergy+	Improper material handling and disposal,^a^ airborne allergens
2. Sharps injury+	Accidental perforations or cuts and improper waste disposal
3. Eye injury+	No protection gear, airborne particulates, and high‐energy light
4. Stress+	High work activity, emotional situations, and loud noises
5. Rhinitis and conjunctivitis+	Inadequate ventilation, disinfectants, aerosols, chemicals, biomaterials,^a^ and waste products
6. Hearing impairment+	High frequency sounds
7. Other	Poison ingestion

*Note.* + indicates a health problem that may apply also to a dental therapy dog, although the ranking of potential health problems for the dental therapy dog is likely to differ from humans.

a Particularly products containing resin monomers, for example, primers, adhesives, composite resins, acrylics, reliners; radiographic solutions; disinfectants; and essential oils, for example, oil of cloves (eugenol).

#### 3.1.1 Allergy

##### Hazards

Many substances used in the dental clinic are hazardous, such as unpolymerised resins and dental amalgam used in operative dentistry or latex or latex additives used in hand gloves as well as several disinfectants.

Airborne allergens are also present and especially if the dental clinic staff do not follow proper material handling protocols or if the dental clinic operatory ventilation is inadequate. The most common hazards in this respect for humans are volatile resin monomers and airborne powder from latex gloves or following extraoral grinding of a base metal alloy containing, for example, nickel, beryllium, cobalt, or chromium. It is reasonable to assume that these airborne allergens are also potentially hazardous for dogs, because recent evidence suggests that underlying causal factors of allergic diseases are likely common for dogs and their owners (Hakanen et al., 2018). Further, the oral and nasal exploratory behaviour of dogs might put them at increased risk of exposure to some hazards. However, allergy to latex and other components is poorly described in dogs.

##### Risk assessment

Dogs develop hypersensitivity and allergy alike the humans to environmental and food allergens, which is manifested often by a gradual development of atopic dermatitis (Kang, Kim, Jang, & Park, 2014).

Although contact allergy is the most prevalent form of allergy amongst dental personnel, the risk that a dental therapy dog may develop contact allergy seems small given the limited contact dogs would have with those materials and limited data suggesting that allergy to latex and other compounds is a relevant concern in dogs. Allergy to latex and other components of concern in dental facilities has not been reported in dogs.

##### Risk minimisation and proposed best practices

- The dental therapy dog should be positioned above the floor level, if possible, because airborne particles ultimately settle on the floor, and given some movements in the dental clinic operatory may become airborne again before resettlement.
- A dental therapy dog should be a dog breed associated with a low propensity for allergy (Bjelland, Dolva, Nødtvedt, & Sævik, 2014) and living in conditions not associated with an increased risk of allergy development (Meury et al., 2011).

#### 3.1.2 Sharps injury

##### Hazards

Sharp instruments can perforate or cut the skin, with a potential risk of acquiring blood or saliva‐borne pathogens, or opportunistic infections caused by the individual's commensal microbiota. Percutaneous injuries are a substantial risk to all clinic staff members and are caused principally by needle sticks (87%), suture needles (23%), and dental instruments (9%; Shah, Merchant, & Dosman, 2006). The prevalence of sharp injuries are probably underreported and confounding factors besides the education level of all clinic staff members are the extent of adherence to good working practices while carelessness and work‐related stress play also a role (Imran, Imran, & Ashley, 2018). Sharp instruments also pose a risk of percutaneous injury to dental therapy dogs if they are able to encounter them.

##### Risk assessment

The risk of a dental therapy dog to experience percutaneous injury in the dental clinic operatory depends on hazardous clean‐up routines of the work area and improper disposal of waste. If sharp instruments are left untidily spread out on a tabletop tray and the clean‐up routine involves bagging all contents with the paper tray cover and carrying away everything for sorting afterwards in a cleaning area, risk may be created for both personnel and dogs. The chances are high that innocuous items such as used burs, unsheathed needles, empty plastic packs, metal matrix bands, and endodontic files may fall to the floor undetected, and the exploratory nature of dogs could result in trauma from biting or ingesting dropped items.

##### Risk minimisation and proposed best practices

- The therapy dog should be trained to not react to drops and not pick up anything off the ground in the dental operatory room, and the dog handler must pay close attention to the dog's behaviour if any instruments plunge during the dental session.
- Avoid a clean‐up practice that encompasses “bagging” the table tray contents with the paper tray cover for subsequent sorting in a cleaning area, as it is hazardous for both the clinic staff and the dental therapy dog.
- Detecting a percutaneous injury on a dog is more difficult than for a clinic staff member, who can instantly communicate the quality and quantity of the damage, observing signs of discomfort, alternatively vigorous licking of an accessible body part may indicate an injury, although detecting the actual perforation of the skin may be difficult. As for sharps injuries experienced by humans, the best procedure is to bleed the wound gently under running water before washing with soap and water and dry the wound followed by protection with a bandage. Given the circumstances, one may also choose to consult with a veterinarian whether the injury warrants further measures.

#### 3.1.3 Eye injury

##### Hazards

High‐velocity particulates that originate from surface grinding and ultrasound or piezoelectric devices can cause damage to the mucosa/conjunctiva of the eyes of clinic staff members nearby the work field and who do not wear personal protection devices.

A beam of very bright light illuminates the intraoral work field, and the clinic staff members are cautious not to shine this light directly into the patient's eyes. Some patients prefer to wear dark glasses to shield the eyes from the bright light.

Additional eye protection is required when extra high‐energy light is used for polymerising resin monomers or when a soft or hard laser is used for any therapeutic or surgical purposes. There is a wide variation of spectral radiant power and illuminance levels generated by different curing devices that are based on, for example, light‐emitting diode, quartz tungsten halogen, plasma arc, or laser. The parameters used to describe the qualities of the different generated lights are perplexing and hence difficult to address in terms of hazard considerations (Price, Ferracane, & Shortall, 2015). Ultraviolet radiation used in the past for initiating polymerisation of resin composites is no longer in use in dentistry. On the other hand, the most effective emission spectrum for curing monomer resins is in the “blue light,” that is, the short‐wavelength region of the light spectrum, which has the highest potential for damaging the eyes.

##### Risk assessment

A dental therapy dog positioned too close to an operation field risks eye damage if the clinical work generates high‐velocity particulates.

Canine and human eyes are reasonably similar concerning size, anatomy, physiology, and pathology (Whiting et al., 2013). All forms of high‐energy lights that are a potential health risk for humans (Price, Labrie, Bruzell, Sliney, & Strassler, 2016 are likely also the same for the dental therapy dog.

##### Risk minimisation and proposed best practices

- Dental personnel and handlers should be cognizant of the position of the dog with respect to light sources.
- The dental therapy dog should be repositioned further away from the operation field if there is a high likelihood of generating high‐velocity particulates that originate from surface grinding and ultrasound or piezoelectric devices.
- The attenuation of the intensity of the light is proportional to the distance from the source. Maintaining a distance between the patient and the dog would seem to be an adequate measure for minimising the risk of eye damage to the dental therapy dog.
- It may also be prudent to shield the dental therapy dog from all types of high‐energy lights. The aetiopathology remain unknown for many ocular diseases amongst dogs, including cataract, congenital stationary night blindness, glaucomas, keratitis, keratoconjunctivitis, retina microdetachment, retinoschisis, and sudden acquired retinal degeneration syndrome (Ledbetter & Gilger, 2013).
- Specially designed protection goggles have been developed for military and police dogs, and they can in theory be fitted with coloured glass that filter out bands of the light spectrum. However, the dog may not find their use very comfortable. Moreover, glasses should be removed when not needed, because the use of shaded glasses may interfere with the possibility of direct eye contact communication between the dental therapy dog and the patient.
- The dental therapy dog handler could use a towel or own hands to cover the dog's eyes when needed, or the therapy dog could be repositioned so that the dog's head is turned away from the dental drill and the light source.

#### 3.1.4 Stress

##### Hazards

Unplanned delays are frequent in dental clinics, which creates periodically a very high work activity. Clinic staff members are also accustomed to experiencing sudden emotional outbursts from the patient because of sudden pain, surprise, or anger. Moreover, uncooperative patients with occasional erratic behaviour require careful management. Stressful incidents also arise when stakeholders are exposed to unexpected loud noise levels generated by high‐vacuum suctioning or resonance from an ultrasound device or by a rotating instrument.

##### Risk assessment

Under nonstressful situations, experienced therapy dogs monitored by an appropriately trained dog handler seldom show physiological or behavioural indicators of stress, fatigue, or exhaustion (Glenk, 2017).

Stressful situations, on the other hand, affect all stakeholders, and behavioural responses are highly individual and can be unpredictable (Chapman, Chipchase, & Bretherton, 2015a; Chapman, Chipchase, & Bretherton, 2015b; Chapman, Chipchase, & Bretherton, 2015c). Not all dogs may be able to remain calm and relaxed in stressful situations. The potential welfare implications in therapy dogs under such stressful work conditions remain currently largely unknown (King, Watters, & Mungre, 2011; McCullough et al., 2018).

Typical first signs of stress are panting, yawning, whining, and avoidance, eventually leading to escape attempts. Lip licking may also be a sign of stress within the dental clinic setting, which in other circumstances may be considered as an appeasement signalling (Firnkes, Bartels, Bidoli, & Erhard, 2017). A dental therapy dog that undergoes an acute stress response during a treatment session risk becoming unusable because the animal will associate the negative experience with the dental clinic operatory and become very reluctant ever to enter a dental clinic.

##### Risk minimisation and proposed best practices

- The dental staff should notify the dog handler of potential situations where stressful situations or outbursts are likely to occur. That could lead to keeping the dog out if it was deemed too high risk, warning the handler so they can be prepared or use a distraction technique on the dog, move the dog so there is lower risk to it or to the patient if the dog reacts (e.g., jumps).
- If the dog handler becomes stressed for whatever reason during a treatment session, he or she should leave the dental clinic operatory together with the dental therapy dog. Dogs can very easily recognise stress amongst humans and will synchronise their behaviour accordingly and especially with the dog owner (Duranton & Gaunet, 2018).
- The dog handler's role is to prevent, recognise, and manage any stress‐associated behaviour, and he or she must consistently work to influence the dog's perception of the environment and minimise stress responses, for example, by mental stimulation. This interaction has been described as social synchrony between the dog handler and the animal (Pirrone, Ripamonti, Garoni, Stradiotti, & Albertini, 2017). The extraordinary sociocognitive abilities of dogs and its underlying neurobiological mechanism of the dog's human‐like social competence are likely complex (Buttner, 2016). One indication of how important it is to capitalise on recognising the coping style of individual dogs and their bidirectional signalling relationship with their handler has been measured, for example, a cognizance of these capabilities provides for superior performance of snow avalanche search teams (Diverio et al., 2017).
- In a dental clinic operatory, the dog handler is continuously required to scrutinise the demeanour of the dog and at first signs of stress to be prepared to rapidly leave the dental clinic operatory to avoid escalating stress that may at worst end up in an acute stress response.
- Adequate testing and training of both the dental therapy dog and the handler, the dog breed, the social synchrony with the dog handler, and his or her ability to minimise interactions that the dog might perceive as threatening in a dental clinic are all essential to reduce risks to both humans and the dental therapy dog. The disposition to anxiety and fear varies amongst dog breeds, which is partly a genetic trait (Bellamy et al., 2018), and partially reinforced by, for example, unethical training methods, isolation, and living under stressful conditions. An awareness of these elements is required when the dentist is considering the potential suitability of allowing a dental therapy dog into a clinical work environment.
- Enough time to rest and recuperate between and after patient interaction is imperative in order to minimise stress both for the dental therapy dog and the dog handler.

#### 3.1.5 Rhinitis and conjunctivitis

##### Hazards

Inadequate clinic ventilation creates hazardous levels of volatile substances arising from disinfection solutions, aerosols, chemicals, biomaterials, and waste products.

Aldehydes are highly effective disinfectants used previously in many dental clinics. However, their use has been discouraged due to allergenic potential and local skin irritation effects. The alternatives containing chloride compounds and alcohols/ethanols have similar propensities, although in much less extent.

##### Risk assessment

Transient, irritative reactions of the eyes and airways have been observed amongst clinic staff members, mostly associated with exposure to volatiles from resin‐based materials, radiographic solutions, chemicals, and biocides. It is likely that a dental therapy dog will be as affected as humans (Windsor & Johnson, 2006).

##### Risk minimisation and proposed best practices

- Consider keeping the dog out of the room when chemicals are used.
- Consider keeping the dog away from freshly disinfected sites where there might be higher concentrations of residual disinfectant.
- Use lower risk disinfectants like accelerated hydrogen peroxide.
- Avoid biocides on spray bottles and adopt disinfection procedures in closed containers with small surface areas and under vacuum and proper ventilation.

#### 3.1.6 Hearing impairment

##### Hazards

Sound levels are reported as decibel sound pressure level (dB SPL) or dBA values within the frequencies between ~30 and 8,000 Hz. dBA is a mathematical adjustment of dB SPL named A‐weighting, to account for the relative loudness across the sound spectrum as perceived by the human ear. In practice, the difference between dB SPL and dBA values are relatively minor and apply only in the low‐frequency range. High sound density can originate from dental devices and patients. Mixing machines, dental turbines, high‐speed handpieces, and ultrasonic devices have each the capacity to generate SPLs well above 90 dB, and vacuum suctioning of cooling water and saliva may create more than 90 dB SPL. Children that begin to cry, for example, prompted by fear or by pain can generate 100–120 dB SPL, and an angry verbal outburst from an adult for the same reasons can reach even higher sound spikes.

Hazards associated with hearing loss is also related to the sound spectrum and the origin of the contentious debate of whether dental clinic staff over time experience hearing loss. A healthy human ear can hear sounds in the frequency range between 20 and 20,000 Hz, whereas dogs can readily perceive high‐frequency sounds up to 45,000 Hz (Heffner, 1983), analogous to other members of the carnivore animal order (Malkemper, Topinka, & Burda, 2015).

Ultrasound scalers for removing calculus operate with a frequency of 25 kHz and emit sound in the range of 70–120 dB SPL. Transient hearing loss can be measured amongst clinicians after use of ultrasound scalers, although the noise that is heard is resonance generated when the tip of the ultrasound scaler contacts the tooth or restoration surface (Chopra, Thomas, Mohan, & Sivaraman, 2016). Also, dental turbines and high‐speed handpieces may generate high levels in the ultrasound frequency band spectra (Sorainen & Rytkönen, 2002).

##### Risk assessment

There is limited published evidence that permanent hearing impairment occurs amongst dentists caused by a noisy work environment (Ma, Wong, & Mak, 2017). However, many share the view, based on sound level measurements and questionnaire responses, that dental clinic staff members are placing their hearing health at risk in a typical daily work environment (Burk & Neitzel, 2016; Myers, John, Kimball, & Fruits, 2016). The use of hearing protectors to attenuate sound is impractical and hence uncommon because the care providers need continuously to monitor the patient for verbal and non‐verbal feedback while rendering the treatment.

In humans, sounds more than 85 dB SPL can damage sensitive structures in the inner ear on one or both sides and cause noise‐induced temporary or permanent hearing loss. Sounds of less than 75 dB SPL are unlikely to cause hearing loss even after prolonged exposure. The higher the dB SBL, the higher the risk for damage. Extremely loud bursts of sound, such as gunshots or explosions, can also rupture the eardrum or damage the bones in the middle ear resulting in an immediate and usually permanent hearing loss (National Institute on Deafness and Other Communication Disorders, 2019).

It is difficult to judge the risks for noise‐induced hearing loss amongst dental therapy dogs because so little has been published about dB SPL above 8,000 Hz in dental clinics. Noise‐induced hearing impairment amongst dogs has not been studied systematically, although dogs confined to kennels with high ambient noise levels appear to develop hearing impairment (Strain, 2012).

For long, researchers have believed that high‐frequency sounds attenuate rapidly and are therefore less detrimental than low‐frequency sounds for humans. It took a diplomatic crisis in Cuba in 2017 to discredit this idea and opening for hectic research activity on the possible effects of ubiquitous ultrasound that originate from many everyday technologies (Fletcher et al., 2018; Yan, Fu, & Xu, 2019). New research findings on the potential adverse effects of high‐frequency sounds on both human and animal hearing will likely surface in the foreseeable future.

##### Risk minimisation and proposed best practices

- As for other physical phenomena, the inverse square law also applies to sound. Hence, the further away the dental therapy dog is from the source of the noise, the lower the risks of hearing impairment.
- If planning for treatment activities that are likely to generate high sounds, it may be prudent to explain to the patient why the dental therapy dog must leave the dental clinic operatory while the particular treatment procedure is ongoing.
- At around 8–10 years of age, the hearing of dogs becomes impaired. The hearing loss is across the entire frequency range, although primarily in the high‐frequency area, just like humans. (Ter Haar, Venker‐van Haagen, van den Brom, van Sluijs, & Smoorenburg, 2008).
- The owner of the therapy dog must consistently consider whether the dog is experiencing a hearing loss. Dogs may become depressed, disoriented, or even aggressive because of difficulties with adapting to such a change. Signs may be dropping ears in spite of sounds, alternatively suddenly raising ears or tilting their head when there are no sounds. Dogs with hearing loss may also experience tinnitus, which often is accompanied by signs of stress, including pacing, frequent barking, or whining.

#### 3.1.7 Other hazards

##### Hazards

Accidental breakages, knocked‐over bottles, solution spills, and leakages on benchtops or floors are instantaneous hazards for all stakeholders, and for a dental therapy dog also dependant on how well the dog has been trained and is being monitored.

##### Risk assessment

Poisoning is possible if a container with a medication or a substance is leaking or knocked over and the contents somehow trickle to the floor. The dental therapy dog may be tempted by the smell and taste to consume the spill out regardless of whether it is innocuous or a schedule one drug or substance.

##### Risk minimisation and proposed best practices

- Regular control of containers, boxes, and bottles containing medication and substances will minimise risks of undetected leakages.
- Keep bottles and containers open only when used and cap or close straight away.
- The dog handler must be able to command the immediate and full attention of the therapy dog and to discontinue any intake of spilt contents from a knocked‐over container.
- Dogs should be trained not to approach or react to dropped items.

### 3.2 Patient behaviour

Hazards
  Patients may accidentally harm the dog due to their action. Different considerations apply whether the patient is a child or an adult, a person with a cognitive impairment, or if the patient has a mental or behavioural disorder.

Risk assessment
  A child's behaviour may be unpredictable; hence, there is a risk that the child's way of behaving may harm the dental therapy dog. Young children may for instance accidentally poke their fingers into the dental therapy dog's eye or pinch the dog.
  Emotionally unstable patients may suddenly turn aggressive, scream, or shout during the dental treatment and thereby frighten the dog. Some dental therapy dogs may be more accustomed to such behaviour and not show signs of stress although the dog may not feel comfortable.
  Elderly patients may have problems with their balance and unintentionally step on the dental therapy dog or even stumble and fall over the dog.
  For all patients, initially anxious patients that become exuberant after having “survived” a dental treatment may physically harm the dental therapy dog by inadvertently in their excitement squeezing the therapy dog too hard.

**Figure 1:**
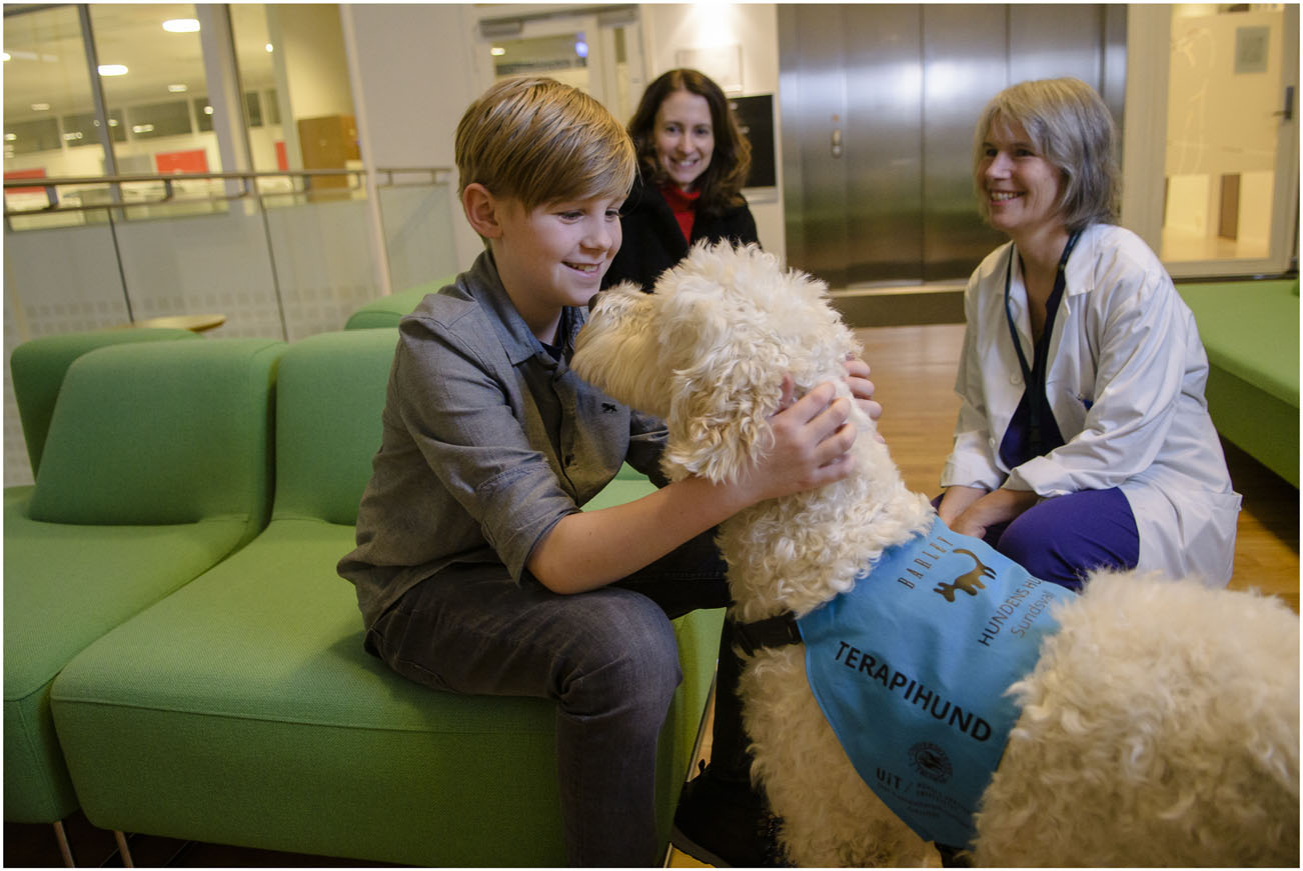
The first contact between the patient and the dental therapy dog should be in the waiting area to establish the necessary building of a relationship between the patient and the dental therapy dog. Photo: L.Aa. Andersen

Risk minimisation and proposed best practices
  • For any single patient, the dentist and the dental therapy dog handler need to consider all possible scenarios concerning a proper risk assessment and to prepare accordingly before bringing the dental therapy dog into the dental clinic.
  • The patient should always first meet the dog handler without the presence of a dental therapy dog. Unless the dog handler is a licensed health professional, he or she cannot inquire about any patient health issues. Still, the dog handler must determine whether there are any perceptible risks to human or animal health in the dental operatory. It may be prudent that the dog handler establishes a standardised approach that include a sequence of specific queries and answers and corresponding actions depending on the responses.
  • The first encounter between the patient and the dental therapy dog should be in the patient waiting area, if feasible, or in a separate consultation room (Figure 1). Patient anxiety escalates when seated in the dental clinic operatory, which may jeopardise the necessary building of a relationship between the patient and the dental therapy dog.
  • During the first encounter between the patient and the dental therapy dog, the dog handler needs to observe and assess their behaviours and determine whether the presence of the dental therapy dog in the dental operatory is not advisable due to risks to human or animal health.
  • The dog handler must during the treatment session be observant and pay attention to early signs of the dog becoming uncomfortable and if deemed necessary, to leave the dental clinic operatory together with the dental therapy dog.

### 3.3 The patient as a disease vector

Hazards
  A patient with a communicable disease may indirectly transmit the pathogen to the therapy dog via aerosols, if the clinic staff contravene the protocols for avoiding cross‐contamination or if the waste management is poor (Sebastiani, Dym, & Kirpalani, 2017). Bacteria, including zoonotic pathogens, can also be exchanged between animals and humans via direct contact (Schwarz, Loeffler, & Kadlec, 2017).
  Aerosols and splatter produced during dental treatments have the potential to spread an infection to operators and patients. Aerosols contain mainly gram‐positive organisms (*Staphylococcus epidermidis* and *Micrococcus* spp.), gram‐positive, rod‐shaped bacteria, and those creating endospore as well as nonspore‐forming bacteria and mould fungi (e.g., *Cladosporium* and *Penicillium*; Kobza, Pastuszka, & Bragoszewska, 2018; Watanabe, Tamaki, Yokota, Matsuyama, & Kokeguchi, 2018).
  Infectious dental waste can contain clinically relevant bacteria with important resistance and biofilm profiles (Laheij, Kistler, Belibasakis, Välimaa, & de Soet, 2012). Proper waste disposal applies both in the actual dental clinic operatory and elsewhere in the vicinity of the dental clinic. Not all dentists provide patients with appropriate postoperative‐advice regarding how to dispose of blood‐contaminated tampons following, for example, tooth extraction, which may end up even on the outdoor pavement (Dai et al., 2016).

Risk assessment
  Most human pathogens are not transmissible to dogs. Hence, the risk of the dog acquiring a communicable disease from a human is low.
  Some patients, mostly children, may put their fingers in their mouth to show the dentist, for instance, a loose tooth or to point at where it hurts and after that continue to pet the dental therapy dog's legs. The dental therapy dog may later groom his or her leg by licking and thereby end up with human bacteria (virus and fungi).
  Microorganisms in waste may be transmitted to dogs (and humans) if the principles of biosafety measures are neglected (Tagliaferri et al., 2017).
  The exposure to the microorganisms identified in aerosols is not considered a significant occupational hazard for dental care professionals (v). However, much remain unknown (Kobza et al., 2018), which include estimations of risks to dental therapy dogs to inhalation of contaminated aerosols from human dental treatment.

Risk minimisation and proposed best practices
  • Universal guidelines regarding hygiene in the dental clinic (CDC, 2018) reduce risk transmission of pathogens for both humans and the dental therapy dog.
  • All persons in direct contact with the dental therapy dog should adopt frequent hand‐hygiene.
  • Avoid the presence of a dental therapy dog if the dental patient has a communicable disease. A patient with a communicable disease in need of any elective dental therapy should postpone further care until he or she is fully recovered.
  • The dog handler must ensure that the dental therapy dog never can reach any contaminated instruments or bio‐waste such as extracted teeth, tampons, or anything else that has been the patient's mouth (e.g., cotton rolls).
  • Grinding that likely cause contaminated debris and aerosols should not be undertaken in the presence of a dental therapy dog.
  • The waste container in the dental clinic operatory must have a lid or be placed in a cabinet to prevent that the dental therapy dog accesses the garbage.
  • A frequent and close monitoring of the health of the dental therapy dog by a veterinarian seems prudent. Currently, there are no data to substantiate how often this should occur and what should be examined beyond a routine physical examination.
  • Dentists must proactively instruct patients how to properly dispose blood‐contaminated tampons following dental surgery without risks to others.

## 4 DISCUSSION

If the patient prefers having the dental therapy dog close, one needs to think about the comfort for both the patient and the dental therapy dog. There needs to be enough space for both. A large dog may be too heavy for a small, tiny person and a little dog may be too fragile if the patient needs to grasp around the dog's body or the dog's leg to feel safe and relaxed during the dental treatment.

Dental patient chairs often have a surface that is slippery. The dental therapy dog may be better secured and feel more relaxed when seated in the chair if the paws are covered with antislip dog socks.

**Figure 2:**
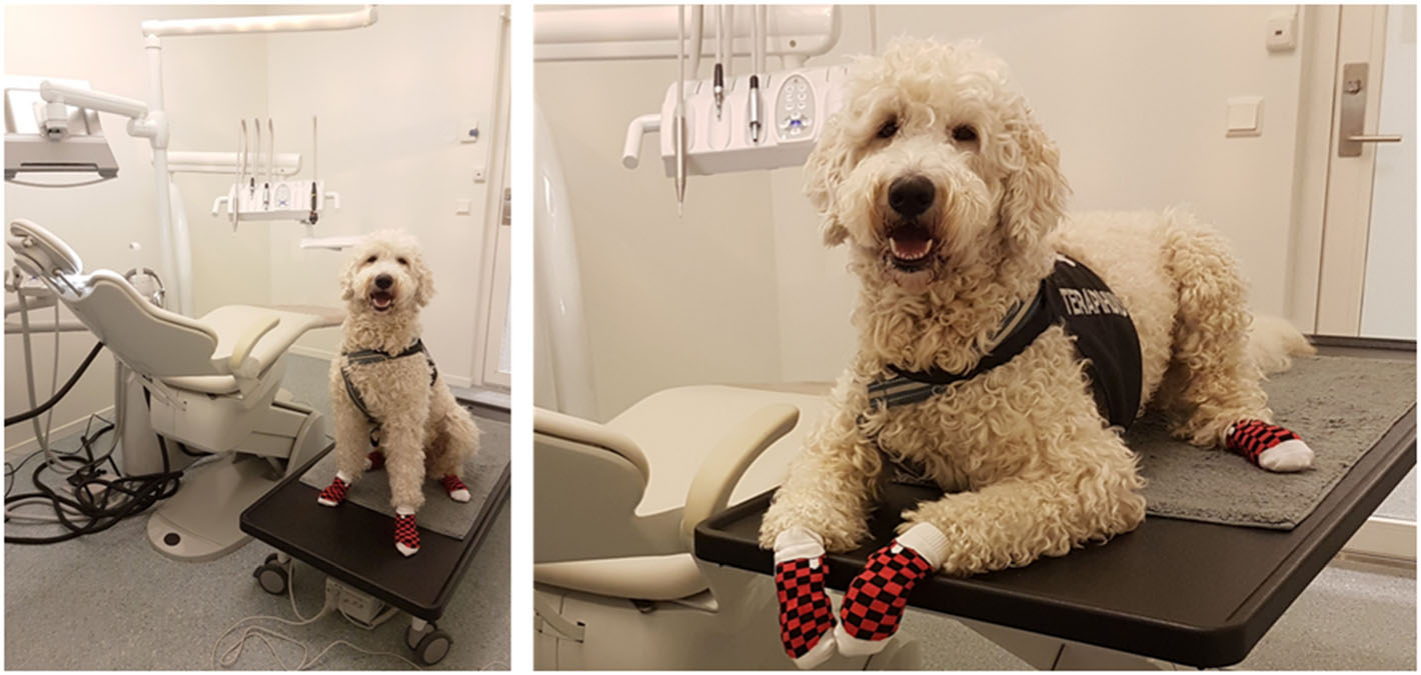
A sturdy and mobile professional veterinary table with wheels that can be securely locked and with adjustable height is ideal. The dental therapy dog needs to be trained to adapt effortlessly to this position arrangement. Photo: A.M. Gussgard

Both the patient and the dental therapy dog may be more comfortable if there is a separate table for the animal beside the dental chair. A sturdy and mobile professional veterinary table with wheels that can be securely locked and with adjustable height is ideal. The dental therapy dog needs to be trained to adapt seamlessly to this position arrangement (Figure 2).

Whether the dental therapy dog is placed in the dental chair together with the patient or on a veterinary table next to the dental chair, both position arrangements will affect the positioning of the dentist's instrument table. Right‐handed dentists, which often keep the instrument table placed on the right side of the patient, may discover that a better position from an ergonomic perspective is to keep the instrument table on the left side of the patient, in other words, further away from the head of the dental therapy dog.

It may not be necessary to retain the dog in the dental chair (or on the veterinary table) during the entire dental appointment. One approach may be that the patient has the dental therapy dog in the dental chair only the first few minutes to calm down during the clinical examination and local anaesthesia. The dental therapy dog can then move elsewhere and perhaps remain on a floor carpet in the corner of the dental clinic operatory. Such an arrangement will reduce the risks of harmful noises and maintain a more distant position away from the hazards described in the previous sections.

There should be no reasons to retain the dental therapy dog inside the dental clinic operatory during the cleaning of the dental clinic operatory between different patients. Bringing the dog to a quiet place after finishing working with the patient enables the dental therapy dog a chance to recuperate. A rested dog presumably has a higher threshold before signs of stress‐activated behaviour due to work overload appear, and the dental clinic operatory can be cleaned without risks of any chemicals or remnants from the dental treatment harming the dental therapy dog.

It is difficult to recommend how often the dental therapy dog should work or how many patients the dental therapy dog is able to cope with each day in a dental clinical setting. Determining factors are patient characteristics (e.g., age and level of dental anxiety), what kind of dental procedure (examination versus advanced treatment), how demanding is the work for the dental therapy dog, and how experienced is the dental therapy dog and the dog handler. Most importantly, each session has to be individually modified, and the dental therapy dog has to be given ample time for recreation. Because both anxiety behaviour and the interventions in dentistry are highly individualised, each treatment session has to be exclusively tailored to that specific appointment and the individual patient. One should always ask “what is the objective by bringing the dental therapy dog into the dental clinic operatory today?”

## 5 CONCLUSIONS

Introducing a dental therapy dog to work with patients in a dental clinical setting creates hazards, but these seems to be manageable given proper training and adherence to established clinical protocols. At the core is an intention to help anxious patients overcome the anxiety so necessary dental care can be completed. The use of a dental therapy dog is an antianxiety approach that may provide the necessary support to overcome anxiety and achieve this objective, thus avoiding the risks associated with use of sedation or general anaesthesia. The hazards in the dental clinics that have been described in this paper are real. However, all the risks for the health and safety of the dental therapy dog that we have identified and appraised are conceivable risks, and currently, there is no scientific data to substantiate whether the rates of the individual risks may be considered as low, medium, or high.

## CONFLICT OF INTEREST

None declared.
